# Current perspectives on the management of refractory or relapsed classic hodgkin lymphoma in brazil: Balancing efficacy, safety, and tolerability

**DOI:** 10.18632/oncotarget.28541

**Published:** 2023-12-12

**Authors:** Flávia Dias Xavier, Danielle Leão Cordeiro de Farias, Abrahão Elias Hallack Neto, Glaciano Nogueira Ribeiro, Marco Aurelio Salvino de Araujo, Thiago Xavier Carneiro, Otavio Cesar Carvalho Guimarães Baiocchi

**Affiliations:** ^1^Hospital Universitário de Brasília-Universidade de Brasília/Ebserh, Brasília, DF, Brazil; ^2^Hospital DF Star, Oncologia D’Or, Rede D’Or, Brasília, DF, Brazil; ^3^Hospital A Beneficência Portuguesa de São Paulo, São Paulo, SP, Brazil; ^4^Universidade Federal de Juiz de Fora, Juiz de Fora, MG, Brazil; ^5^Clínica Hematológica/Grupo Oncoclinicas, Belo Horizonte, MG, Brazil; ^6^Universidade Federal da Bahia, Salvador, BA, Brazil; ^7^Instituto D'Or de Pesquisa e Ensino, IDOR, BA, Brazil; ^8^Universidade Estadual do Pará, Belém, PA, Brazil; ^9^Universidade Federal de São Paulo, São Paulo, SP, Brazil; ^10^Hospital Alemão Oswaldo Cruz, São Paulo, SP, Brazil

**Keywords:** brentuximab vedotin, drug therapy, hodgkin lymphoma, nivolumab, pembrolizumab

## Abstract

Classic Hodgkin lymphoma (CHL), which accounts for 90–95% of all cases of Hodgkin lymphoma, is the most frequent cancer in adolescents and the most frequent lymphoma in adolescents and young adults. Despite progressive improvements over past decades and the general sensitivity of CHL to frontline chemotherapy, approximately 10–15% of patients have refractory disease that either does not respond to such therapy or progresses after an initial partial response. In patients with refractory or relapsed disease, standard treatment until recently consisted mainly of salvage chemotherapy, in many cases followed by high-dose chemotherapy and autologous stem-cell transplantation. However, improved understanding of the pathobiology of CHL, coupled with the introduction of novel agents, has markedly changed the treatment landscape in the past decade. Although refractory or relapsed CHL continues to be challenging, the therapeutic landscape is undergoing profound changes brought about by novel agents, particularly brentuximab vedotin and immunotherapy. In this review, we discuss the most salient treatment options for adult patients with refractory or relapsed CHL, with a special focus on the Brazilian healthcare setting, which is constrained by inherent characteristics of this system. In the attempt to balance efficacy, safety and tolerability, practicing physicians must rely on clinical trials and on results from real-world studies, and use their own point of view and experience, as well as patient characteristics and previous therapy, to make treatment decisions for refractory or relapsed CHL.

## INTRODUCTION

Classic Hodgkin lymphoma (CHL) accounts for 90–95% of all cases of Hodgkin lymphoma (HL), with the remaining cases being currently classified as nodular lymphocyte-predominant HL [1–3]. CHL, which is divided into four subtypes with somewhat distinct epidemiological features, is very rare before 12 years of age, but is the most frequent cancer in adolescents and the most frequent lymphoma in adolescents and young adults, with a second peak in late life for certain subtypes [1, 4]. CHL most often involves cervical lymph nodes and/or the mediastinal, axillary and para-aortic regions, and 50–60% of patients have early-stage disease (stage I or II) at diagnosis [1, 3, 5]. Approximately 10–15% of patients have refractory disease that either does not respond to initial therapy or progresses after an initial partial response; moreover, relapse may occur in 10–15% of patients with favorable prognosis, early stages (I or II) and in 15–30% of patients with more advanced disease [6–9]. Although 80–90% of newly diagnosed patients can be cured when treated with contemporary frontline therapy, CHL is primarily refractory or recurs in nearly 10% of patients with early-stage disease and up to 30% of those with advanced (stage III or IV) disease [6–13]. Primary refractoriness is adequately defined either by progression during chemotherapy or radiotherapy at any time up to 3 months after the end of frontline treatment or by persistence of substantial residual uptake on positron-emission tomography (PET) using the quantitative 5-point-scale Deauville score [4]. Nevertheless, primary refractoriness is also defined more simply by progression at any time up to 3 months after the end of frontline treatment, by lack of complete response (CR) or lack of CR or partial response (PR) to such treatment, or by persistence of substantial residual uptake on PET scanning [14, 15]. Additionally, a distinction can be made between early relapse (3 to 12 months from frontline treatment) and late relapse (>12 months) [14, 15]. As a general rule, response assessment in CHL follows the Lugano criteria [16].

In patients with refractory or relapsed HL, standard treatment until recently consisted mainly of salvage chemotherapy, in many cases followed by high-dose chemotherapy and autologous stem-cell transplantation (ASCT) [17–24]. However, improved understanding of the pathobiology of CHL, coupled with the introduction of novel agents, has markedly changed the treatment landscape in the past decade [4, 24–31]. In this article, we discuss the management of adult patients with refractory or relapsed CHL, with a special focus on the Brazilian healthcare setting and in an attempt to assess the most salient issues relating to efficacy, safety and tolerability of salvage therapy.

## EPIDEMIOLOGY AND CLINICAL FEATURES OF CHL IN BRAZIL

In several countries, HL represents 10–15% of all cases of lymphomas [4, 32, 33]. In Brazil, nationwide estimates for 2020 were of nearly 12,000 new cases of non-Hodgkin lymphomas and a total of nearly 2,600 cases of HL every year; mortality data do not appear to have been updated recently, but in 2017 it was estimated that crude rates were 0.35/100 thousand men and 0.25/100 thousand women, with a total of 355 yearly deaths [34]. Since 2009, countrywide information on HL has been collected by the Brazilian Prospective Hodgkin’s Lymphoma Registry (NCT02589548), which was implemented to gather data on the sociodemographic and clinical features, as well as treatment modalities and outcomes for patients with HL aged 12 years and older [3, 35].

The first results of the registry concern 674 patients with CHL analyzed out of a total of 756 patients registered from January 2009 to 2014 [3]. The key features of the 674 patients with CHL are shown in Table 1. Moreover, extranodal involvement was present in 32% of patients, and bulky mediastinal disease in 28%. No results were shown for immunohistochemistry, but it is well known that nearly 100% of cases of CHL express CD30 [1, 4]. Of note, the prevalence of advanced disease and adverse prognostic features (65%) was higher than in many case series [1, 5].

**Table 1:** Selected characteristics of 674 patients with CHL, according to Biasoli et al. [3]

Characteristics	*N* (%)
Female sex	342 (51)
Age <60 years	610 (91)
Eastern Cooperative Oncology Group performance status <2	585 (87)
Ann Arbor stage	
I	5 (<1)
II	300 (45)
III	152 (23)
IV	204 (30)
Missing	3 (<1)
Risk group^*^	
Early favorable	44 (6)
Early unfavorable	178 (26)
Advanced	437 (65)
Missing	15 (1)
Presence of B symptoms	463 (69)
Histological subtype	
Nodular sclerosis	528 (78)
Mixed cellularity	86 (13)
Lymphocyte-rich	11 (2)
Lymphocyte-depleted	9 (1)
Classical HL unclassified	40 (6)

^*^According to the German Hodgkin Study Group risk group classification [41].

## FRONTLINE TREATMENT IN BRAZIL

Decades of clinical research have established the efficacy and general safety of frontline treatment for patients with HL [4]. The key phase 3 trials with doxorubicin, bleomycin, vinblastine and dacarbazine (ABVD) in stage III/IV disease have shown rates of progression-free survival (PFS) and overall survival (OS) at 3 years of 75% and 90%, respectively [8], or similar rates (of 76% and 90%, respectively) for 5-year PFS and OS among all patients (i.e., including unfavorable stage I/II disease) [10]. In another phase 3 trial, results according to stage (I/II vs. III/IV) were failure-free survival rates of 82% vs. 71% at 3 years and 82% vs. 67% at 5 years, respectively; OS rates according to stage were 94% vs. 85% at 5 years [9]. Specifically for unfavorable stage I/II disease treated with four or six cycles of ABVD plus involved-field radiotherapy, the 5-year event-free survival was 85.9% or 89.9%, with 5-year OS of 94% or 93%, respectively (the corresponding rates were 88.8% and 93% for bleomycin, etoposide, doxorubicin, cyclophosphamide, vincristine, procarbazine and prednisone [BEACOPP]) [13]. Finally, among patients with stage IIB, III, or IV, or an international prognostic score of ≥3, the 7-year rates of event-free survival were 78% among patients randomized to BEACOPP and 71% among those randomized to ABVD, with 7-year OS rates of 89% and 84%, respectively [22].

Although patients and researchers from Brazil increasingly participate in international clinical trials, and notwithstanding many publications on basic and correlative science in HL, to our knowledge there are scant published results on treatment outcomes from local trials with older treatment regimens [36]. Likewise, case series are scarce and present results that are less relevant in light of current standards of care for newly diagnosed patients [37–39]. Nevertheless, results from the Brazilian Prospective Hodgkin’s Lymphoma Registry are a valuable source of information on frontline treatment patterns in this country. The analysis of 674 patients with CHL registered from January 2009 to 2014 has shown a median time from diagnosis to treatment initiation of 21 days, not unlike the median reported from the US National Cancer Database for patients diagnosed between 1998 and 2011 (26 days) [40]. Of note, the median time from the onset of symptoms to diagnosis was 6 months. Regarding treatment patterns, 93% of the 674 patients received ABVD as frontline treatment, and the remaining were treated with standard or escalated BEACOPP or other regimens. Radiotherapy was added to ABVD in 33% of the patients with advanced disease, in 65% of those with early unfavorable disease, and in 77% of those with early favorable disease, as ascertained using the German Hodgkin Study Group (GHSG) risk classification [41]. This was involved-field radiotherapy in 80% of patients thus treated and extended-field radiotherapy in the remaining 20% [3]. With regard to overall treatment toxicity, 17 (2.5%) patients died from complications during frontline treatment; this rate is higher than that typically reported in large clinical trials (<1.0%) [9, 10], even though a rate of 2% has also been reported [8].

Regarding treatment outcomes, the 3-year PFS rates in early favorable, early unfavorable, and advanced disease were 95%, 88%, and 66%, respectively. Corresponding OS estimates at 3 years were 100%, 96%, and 86%, respectively. With a few exceptions, such PFS and OS results are somewhat inferior to some of those reported from the large clinical trials discussed above [8–10, 13, 22]. Moreover, analysis of PFS and OS according to socioeconomic status (SES) has disclosed statistically significant differences favoring higher versus lower SES [35], thus echoing findings from Brazil and other countries in HL and other cancer types [42–47]. These results suggest considerable room for improvement, particularly in light of the fact that registered patients are likely to represent those seen in referral centers, and given the association between hospital volume and outcomes in HL [48]; in other words, results may be worse among Brazilian patients with CHL not participating in the registry.

Given the availability of novel agents with activity in refractory or relapsed CHL [14, 15, 24, 26, 29] (discussed in more detail below regarding their role in refractory and relapsed disease), the standard of care for frontline therapy is subject to change soon. For example, brentuximab vedotin (BV), an anti-CD30 antibody conjugated with the cytotoxic agent monomethyl auristatin E, can be safely combined with doxorubicin, vinblastine and dacarbazine (AVD), although the evidence so far suggests that it should not be combined with ABVD due to a potentiation of the risk of pulmonary toxicity from bleomycin [49]. In a phase 3 trial, BV was combined with AVD and compared with ABVD, showing an improvement in PFS when used as frontline therapy in patients with advanced CHL [25, 50]. Although modified PFS was the primary endpoint in that trial [41], recently data from a median of 6 years of follow-up showed both PFS and OS advantage of BV-AVD over those who received ABVD [51]. Since patients with CHL often benefit from second- and third-line treatments, OS gains can only be demonstrated after long-term follow-up. As a result, PFS is generally accepted as a surrogate endpoint in CHL. Nevertheless, BV—in combination with AVD—is already considered a frontline option in some guidelines [52], and is approved for this indication in several countries, including Brazil (for the latter, in stage IV). Other novel agents that are being investigated for frontline treatment of advanced CHL are the anti-programmed death 1 (PD-1) antibodies, nivolumab and pembrolizumab. Nivolumab was combined in a concomitant or sequential fashion with AVD in phase 2 trials, and early results have been encouraging both in early-stage, unfavorable CHL [53] and among patients with more advanced disease [54]. Likewise, pembrolizumab combined sequentially with AVD was tested in a single-arm phase 2 trial, with promising early results in early unfavorable and advanced-stage disease [55]. There are currently no published data on the use of these novel agents for newly diagnosed patients in Brazil, where these monoclonal antibodies have a clear niche in refractory and relapsed disease, as discussed below.

## MANAGEMENT OF REFRACTORY AND RELAPSED DISEASE

### Chemotherapy and targeted therapy with older agents

Chemotherapy has been the mainstay of treatment for HL for nearly six decades [56], and the current chemotherapy standards of ABVD and BEACOPP—depending on risk stratification—continue to play a key role in frontline therapy, notwithstanding the increasing addition of targeted therapy and/or immunotherapy to the therapeutic arsenal, or even to the possibility of chemotherapy-free regimens in the future. In Brazil, procarbazine is not currently available, thus influencing the choice of frontline chemotherapy. Patients with refractory or relapsed CHL are still treated with curative intent, typically with one of a variety of salvage chemotherapy regimens, which is usually followed by response consolidation through the use of ASCT in patients who are eligible to this procedure [4, 17, 18, 24, 26]. For patients who have already received an ASCT or who are not eligible because of age or comorbidities, an individualized approach is often recommended, with sequential use of conventional or novel agents, or with participation in clinical trials [41]. Although the chemotherapy agent bendamustine, and the older targeted agents, lenalidomide and everolimus, have been assessed as single agents or in combinations as treatment for refractory or relapsed HL in phase 2 trials, they are not widely in this era of novel targeted agents and immunotherapy, discussed below [24, 41]. Nevertheless, these older agents are listed as treatment options in the National Comprehensive Cancer Network guidelines (although not approved for HL in the US) [52], and can certainly be used in selected cases [4, 26].

### The expanding niche for novel agents

As several other hematologic malignancies, CHL consistently expresses antigens that may serve as specific targets for monoclonal antibodies; foremost among these targets for its ubiquity and relevance in CHL is CD30. The antibody BV, which targets CD30, was initially used in patients with advanced HL with a relapse after ASCT and a median of 3.5 prior lines (range, one to 13 regimens). In a pivotal, single-arm phase 2 trial enrolling 102 patients, the response rate was 75% (with CR in 34% of patients), with acceptable toxicity [57]. Importantly, prolonged follow-up of those patients disclosed a median PFS of 9.3 months in the overall population, and the 34 patients with a CR had 3-year OS and PFS rates of 73% and 58%, respectively; moreover, 47% of patients with a CR (i.e., 34 of 102 [16%] of the total) remained progression-free after a median of 53 months of follow-up [58]. After 5 years of follow-up, median OS and PFS had not been reached in the 34 patients achieving CR; moreover, 13 of these patients remained relapse-free longer than 5 years and may have been cured (four of these patients with the help of a subsequent allogeneic transplantation) [59]. A smaller phase 2 trial among patients with a relapse after allogeneic transplantation showed a response rate of 50% (CR of 38%) and a median PFS of 7.8 months [60]. Interestingly, retreatment of patients with a previous CR or PR to BV led to a response rate of 60% (30% CR) [61].

Results from these and other trials led to the design of a phase 3 trial comparing BV versus placebo as consolidation for patients undergoing ASCT with a high risk of relapse (Table 2) [14]. High risk was defined as primary refractoriness, relapse after frontline therapy with an initial remission of less than 12 months, or extranodal involvement at the start of salvage chemotherapy before ASCT. Treatment consisted of 16 cycles of BV, starting 30–45 days after ASCT. Following the positive early results with regard to PFS, the primary endpoint in the trial [14], updated results showed 5-year PFS rates of 59% with BV and 41% with placebo (hazard ratio of 0.52) [62]. Moreover, upfront consolidation with BV significantly delayed time to subsequent therapy. These phase 2 and 3 trials led to the approval of single-agent BV for the treatment of adult patients with CHL or CD30-positive HL in several countries and in different refractory/relapsed settings. Of note, BV is widely used in Brazil—depending on local reimbursement issues—and can be safely combined with other agents used among these patients, such as bendamustine [63–65] and multi-agent chemotherapy [66–68], thus providing enhanced treatment options in preparation for ASCT.

**Table 2:** Selected results from clinical trials or trial cohorts among patients with refractory or relapsed CHL

Agent	First author(s)	Setting	N^*^	Overall RR	CR rate	PFS	Selected safety results^**^	Discontinuation due to adverse events
BV	Moskowitz, et al. 2003 [14] Moskowitz, et al. 2018 [62]	Consolidation after ASCT	165	NA	NA	59% at 5 years	Treatment-emergent peripheral neuropathy, 67%^†^ Neutropenia of any grade, 35% Treatment-emergent pulmonary toxicity, 5%	33%
BV	Younes, et al. 2012 [57] Gopal, et al. 2015 [58] Chen, et al. 2016 [59]	Relapse after ASCT	102	75%	34%	22% at 5 years	Peripheral sensory neuropathy, 42% Neutropenia, 19%	20%
BV	Kuruvilla, et al. 2021 [15]	Relapse after/ineligible to ASCT	153	54%	24%	8 months (median PFS)	Peripheral neuropathy of any grade, 13% Neutropenia of any grade, 10%	16%
BV	Gopal, et al. 2012 [60]	Relapse after AlloSCT	25	50%	38%	7.8 months (median PFS)	Peripheral sensory neuropathy, 48% Neutropenia of any grade, 28%	36%
Nivolumab	Armand, et al. 2018 [75]	No prior BV	63	65%	29%	18 months (median)	Not reported separately for this cohort, but overall in the trial, the most common events of any grade were diarrhea (35%) and fatigue (35%)	5%
Nivolumab	Armand, et al. 2018 [75]	Relapse after post-ASCT BV	80	68%	13%	15 months (median)	Not reported separately for this cohort, but overall in the trial, the most common events of any grade were diarrhea (35%) and fatigue (35%)	11%
Nivolumab	Armand, et al. 2018 [75]	Relapse after BV before or after ASCT	100	73%	12%	12 months (median)	Not reported separately for this cohort, but overall in the trial, the most common events of any grade were diarrhea (35%) and fatigue (35%)	7%
Nivolumab	Younes, et al. 2016 [77]	Relapse after ASCT and BV	80	66%	9%	NA	The most common events of any grade were and fatigue (36%), pyrexia (31%), and diarrhea (26%)	4%
Pembrolizumab	Armand, et al. 2016 [72]	Relapse after BV (prior ASCT in 71%)	31	65%	16%	46% at 1 year	The most common treatment-related adverse events were hypothyroidism (16%), diarrhea (16%), nausea (13%), and pneumonitis (10%)	7%
Pembrolizumab	Chen, et al. 2017 [73]	Relapse after post-ASCT BV	69	74%	22%	63% at 9 months for 3 cohorts	Not reported separately for this cohort, but overall in the trial, most relevant events of any grade were fever (24%), diarrhea (17%), hypothyroidism (14%)	5.8%
Pembrolizumab	Chen, et al. 2017 [73]	Relapse after BV, ASCT-ineligible	81	64%	25%	63% at 9 months for 3 cohorts	Not reported separately for this cohort, but overall in the trial, most relevant events of any grade were fever (24%), diarrhea (17%), hypothyroidism (14%)	3.7%
Pembrolizumab	Chen, et al. 2017 [73]	Relapse after ASCT (prior BV in 42%)	60	70%	20%	63% at 9 months for 3 cohorts	Not reported separately for this cohort, but overall in the trial, most relevant events of any grade were fever (24%), diarrhea (17%), hypothyroidism (14%)	3.3%
Pembrolizumab	Armand, et al. 2019 [76]	Consolidation after ASCT	30	NA	NA	81% at 19 months	Only treatment-related events reported: grade 2–3 transaminitis, 17%; grade 4 neutropenia, 3%; grade 2-3 diarrhea/colitis, 10%; grade 2 hypothyroidism, 3%	16%
Pembrolizumab	Kuruvilla, et al. 2021 [15]	Relapse after (37%)/ineligible to (63%) ASCT	151	66%	25%	13 months (median PFS)	Hypothyroidism of any grade, 16% Pyrexia of any grade, 13% Diarrhea of any grade, 9%	13%

Abbreviations: AlloSCT: allogeneic stem-cell transplantation; ASCT: autologous stem-cell transplantation; BV: brentuximab vedotin; CR: complete response; NA: not applicable or not yet available; PFS: progression-free survival; RR: response rate. The ~ sign indicate approximate rates read from published Kaplan-Meier curves. ^*^Considering patients with the novel agent of interest. ^**^Reported terms. ^†^Versus 19% with placebo, both rates using a standardized Medical Dictionary for Regulatory Activities query.

The combination of BV and bendamustine has been the subject of several phase 2 trials and observational studies for salvage therapy. In a phase 2 trial involving 40 patients with refractory or relapsed disease, a complete metabolic response was observed in 78.9% of 38 evaluable patients; the response rate was 75.0% in the primary-refractory subset, and 94.4% among patients with relapsed disease. The 3-year PFS and OS rates were 67.3% and 88.1%, respectively [63]. In a phase 2 trial among 55 patients (28 with primary refractory and 27 with relapsed disease), the response rate after a median of two cycles of BV combined with bendamustine was 92.5% (73.6% CRs) [65]. Updated results from this trial showed 3-year PFS and OS rates of 60% and 92%, respectively [69]. In an observational study from the Mayo Clinic involving 207 patients with refractory or relapsed disease eligible to ASCT and treated with a variety of salvage regimens, those treated with BV plus bendamustine had significantly higher overall and CR rates as first salvage therapy, and a larger number of patients were bridged to transplantation after BV plus bendamustine than after ifosfamide, carboplatin and etoposide, leading the authors to conclude that the former combination may be preferable to the latter [70]. Finally, in third or subsequent lines, the combination of BV and bendamustine led to a response rate of 79% (CR in 62%) among 30 patients treated in the real-life setting [71].

After nearly a century of unfulfilled promise, immunotherapy has finally come of age and taken center stage in cancer therapy, mostly due to the activity of immune checkpoint inhibitors (CPIs) and adoptive cell therapy, particularly chimeric antigen receptor T-cells. Over the past few years, HL has also become a beneficiary of such developments. Nivolumab and pembrolizumab are both CPIs targeting PD-1 and with single-agent activity in HL. This activity has been demonstrated in the setting of relapsed disease after BV in patients ineligible to ASCT [72, 73], after ASCT [73–76], after ASCT and BV [72–77], and even after allogeneic transplantation (with previous BV in all cases) [78]. These trials led to the approval of both nivolumab and pembrolizumab in several countries, including Brazil, for the treatment of patients with refractory or relapsed CHL (Table 2). Moreover, nivolumab can be safely combined with BV, and such a combination produced a 3-year PFS rate of 77% as first salvage therapy in patients with refractory or relapsed CHL (91% among patients undergoing ASCT directly after study treatment) [79]. Recently, interim results from a phase 3 trial comparing pembrolizumab (*N* = 151) versus BV (*N* = 153) among a total of 304 patients with refractory or relapsed CHL have been published [15]. These patients were ineligible (63%) for or had relapsed after ASCT (37%), and 5% had received prior BV therapy. The CR rate was similar for both agents (25% versus 24%), but the overall response rate was nominally—although not statistically—higher for pembrolizumab (66%) than for BV (54%). After a median follow-up of 26 months, the median PFS was approximately 13 months with pembrolizumab and 8 months for BV (hazard ratio of 0.65). One treatment-related death from pneumonia occurred in the pembrolizumab arm, but the frequency of adverse events overall was similar in both arms, notwithstanding qualitative differences expected from these two agents (Table 2). These interim results await confirmation and may eventually influence the treatment algorithm for patients with refractory or relapsed CHL, depending on available and emerging data on the role of BV and CPIs in the first line [50, 52].

### Real-life experience with approved novel agents

The assessment of treatment outcomes in real life can also provide useful information on the effectiveness, safety and tolerability of novel agents, thus helping define their utility in clinical practice. There are many publications, most of which from European academic institutions, assessing outcomes outside of the clinical-trial setting. Many of these studies, not all of which summarized below, were included in a meta-analysis of 32 observational reports of single-agent BV in refractory or relapsed CHL reported recently [80]. The authors found pooled overall and CR rates of 63% and 33%, respectively, which are within the ranges reported in the clinical trials displayed in Table 2. Likewise, 1-year (range, 52% to 63%), 2-year (45% to 56%), and 5-year (32% to 33%) PFS, and 1-year (68% to 83%), 2-year (58% to 82%), and 5-year (58% to 62.0%) OS compared favorably with the results from clinical trials.

Individual studies on single-agent BV had sample sizes ranging from 53 to 509 patients, and some included CD30-positive HL rather than CHL. In general, the real-life response rates and tolerability to BV were similar to those reported in clinical trials. For example, the experience in 60 countries with the Named Patient Program showed overall and CR rates of 58–80% and 10–40%, respectively, with PFS and OS results comparable to those from clinical trials [81, 82]. Moreover, several studies have reported prolonged disease control among responding patients [83, 84]. Results consistent with those from clinical trials have also been reported from the Czech Republic and Slovakia, where 58 patients had overall and CR rates of 47% and 33%, respectively, and 1-year, 2-year and 3-year OS rates from initiation of BV of 78%, 62%, and 41%, respectively [85]. Specifically in elderly patients or transplant-ineligible patients, real-life studies have also confirmed the effectiveness of BV, with response rates of 68% to 74% [84, 86]. Moreover, among 509 patients from France, Germany, Italy, Spain, and the UK with a mean age of 46 years, 73% of whom receiving second-line therapy for a first relapse and 44% undergoing ASCT, reported findings broadly consistent with those from guidelines [87]. In a series from Italy, 45 patients were treated with BV as bridge to transplant, whether autologous or allogeneic [88]. Ten of 16 transplant-naïve patients received ASCT, with 50% in CR before transplantation. Among 29 patients treated with BV as bridge to allogeneic transplantation, overall and CR rates were 62% and 24%, respectively, and 93% of them proceeded to transplantation. This and other real-life studies indicate that BV may allow for disease control before transplantation, potentially improving post-transplantation outcomes, also in refractory and heavily pretreated patients, with acceptable tolerability and no significant overlapping toxicities with prior therapies [88–92].

Specifically in the post-ASCT consolidation setting, results have been reported from Turkey and from Italy. In Turkey, 75 patients were analyzed at a median follow-up of 26 months, and 50 patients had an ongoing response (CR in 41 cases), for 2-year PFS and OS rates of 68% and 88%, respectively [93]. In Italy, 105 patients with CHL (both naïve and previously exposed to BV) were analyzed at a median follow-up of 20 months, and the 3-year PFS and OS rates were 62% and 86%, respectively, once again confirming the real-life activity of BV [94]. Although most observational studies were not comparative, a retrospective comparison between BV and chemotherapy in 312 patients from the UK and Germany with a relapse after ASCT (196 treated with BV) showed a median PFS of 27 months for BV, versus 13 months for chemotherapy, with longer 1-year OS rate for BV (78% versus 66%) [95].

Regarding safety and tolerability, real-life studies have also reported results consistent with those from clinical trials, but arguably with more variability, likely as a result of different standards for collection of reporting of adverse events. In the recent meta-analysis, the most common adverse events during single-agent BV were neutropenia (13–23%), anemia (9–39%), thrombocytopenia (4–5%), and grade ≥3 peripheral neuropathy (3–7%), leading the authors to conclude that these results support the safety of BV in the real-life setting [80]. Individual studies have variously reported rates of peripheral neuropathy ranging from 9% to 50% [86, 88, 92, 93, 95], grade 3/4 neurologic toxicity of 6% [81], and neutropenia from 10% to 29% [88, 92, 93]. Treatment discontinuation due to toxicity has been reported in 5–16% of patients [81, 93]. In general, the authors of these studies concluded that real-life treatment with single-agent BV is well tolerated and associated with a tolerability profile consistent with that from clinical trials [82, 84, 92, 93].

Given their later introduction for the treatment of refractory or relapsed CHL, in comparison with BV, currently there are fewer published studies on the real-life experience with nivolumab or pembrolizumab. For nivolumab, investigators from Turkey reported in 82 patients with refractory or relapsed CHL treated with nivolumab in a Named Patient Program [96, 97]. With a median follow-up of 29 months, the overall and CR rates were 70% and 36%, respectively, with an acceptable safety profile; only nine patients discontinued nivolumab due to serious adverse events, and the 2-year PFS and OS rates were 56% and 79%, respectively [97]. The authors concluded that nivolumab is efficacious among patients previously treated with BV, and that it may serve as a bridge to transplantation [96], thus echoing the opinion expressed by others, despite the potential for immune-mediated complications associated with allogeneic transplantation after nivolumab or pembrolizumab [98]. Investigators from Spain reported on 74 patients treated with nivolumab, with overall and CR rates of 58% and 31%, respectively, and a 2-year OS rate of 52%. Treatment-related adverse events were reported in 57% of patients (grade ≥3 in 9%). The authors concluded that the activity and safety of nivolumab were comparable to those reported in clinical trials [99]. For pembrolizumab, real-life data come from a US study including 53 patients with CHL treated with this agent or with nivolumab. The combined overall and CR were 68% and 45%, respectively, and 1-year PFS and OS rates were 75%and 89%, respectively. Importantly, the toxicity was similar to that described in clinical trials [100]. Finally, the GHSG has recently presented results in abstract form relating to 58 CHL patients with a median age of 48 years treated with an anti-PD-1 antibody [101]. Most patients had previous BV therapy (86%) or ASCT (62%). Overall and CR rates were 67% and 20%, respectively, and 2-year PFS and OS rates were 38% and 79%, respectively. Grade 3/4 treatment-related toxicities were reported in 32% of patients. Once again, these results resemble those from clinical trials.

## CURRENT PROSPECTS AND EXPERIENCE IN BRAZIL

### Published experience with transplantation

The published literature on the management of refractory or relapsed adult patients in Brazil is relatively scarce, with the exception of retrospective series on ASCT [102–106]. Based on 694 patients undergoing frontline therapy, investigators from the University of Sao Paulo reported on 188 CHL patients with refractory or relapsed CHL, 107 of whom receiving ASCT from 1995 to 2014 [102]. Primary refractoriness was defined as less than PR at the end of frontline chemotherapy and was present in 99 (14% of the total) patients, whereas relapsed disease—defined as a relapsed after achieving a CR for at least 3 months—was present in 89 (13% of the total) patients. Selected characteristics of patients undergoing ASCT are summarized in Table 3. After ASCT, 90 patients (84%) were in CR, 13 (12%) were refractory; after a median follow-up of 6.7 years, 5-year PFS and OS rates were 60% and 74%, respectively, results within the ranges reported in the literature [17, 18, 20, 107, 108]. Four (4%) patients died of transplant-related mortality, a rate that is on the upper end of the range reported in other series [20, 107, 108]. Factors significantly associated with both PFS and OS were a single line of salvage therapy before ASCT (versus more than one) and a CR before ASCT [102]. The authors concluded that ASCT is efficacious and safe of in the treatment of refractory and relapsed CHL at a large public cancer center in Brazil, and that novel agents, including BV and CPIs are likely to improve transplantation outcomes in in the near future. A second case series from the same university hospital provides insights on the use of nivolumab after ASCT [103]. Of 171 patients with CHL treated between 2015 and 2019, 25 (16%) had primary refractoriness, and an additional 15 patients relapsed after a CR. A total of 24 among these 40 patients underwent ASCT, but among the other patients who were ineligible or did not receive ASCT due to the lack of CR to salvage therapy, five received nivolumab; all five patients could then receive ASCT and were in CR at the time of reporting, with no use of consolidation therapy. A third series comes from another university center and concerns 52 patients undergoing ASCT in Fortaleza, Brazil, between 2009 and 2015, with selected patient characteristics also shown in Table 3 [104]. After ASCT, 81% of patients were in CR, and 5-year PFS and OS rates were approximately 58% and 85%, respectively. Finally, a series of 77 patients with HL considered for ASCT between 1998 and 2006 at one of three centers in Brazil, 53 of whom actually underwent autografting [106]. Table 3 displays selected characteristics of the overall sample of 77 patients. The results showed a higher transplant-related mortality (10% of 77 patients), with 5-year PFS and OS rates of approximately 35% and 55%, respectively. These results compare unfavorably with those from the other series from Brazil and other countries, and the reasons for these findings remain unclear [17, 18, 20, 102, 104, 107, 108].

**Table 3:** Selected characteristics of patients with CHL treated with autologous stem-cell transplantation in Brazil

Characteristics	Series		
	Fatobene et al. [102] (*N* = 107)	Duarte et al. [104] (*N* = 54)	Duarte et al. [106] (*N* = 77)
	*N* (%) or mean	*N* (%) or mean	*N* (%) or median
Year of ASCT	1995 to 2014	2009 to 2015	1998 to 2006
Female sex	61 (57)	22 (41)	31 (40)
Age at diagnosis	26 years	28 years	23 years
Ann Arbor stage at diagnosis			
I/II	47 (44)	29 (56)	27 (35)
III/IV	59 (55)	19 (37)	50 (65)
Missing	1 (1)	4 (8)	0
B symptoms at diagnosis	83 (78)	34 (65)	55 (71)
Histological subtype			
Nodular sclerosis	82 (77)	46 (89)	51 (66)
Mixed cellularity	8 (8)	1 (2)	20 (26)
Lymphocyte-rich	2 (2)	1 (2)	1 (1)
Lymphocyte-depleted	2 (2)	2 (4)	5 (7)
Classical HL unclassified	13 (12)	2 (4)	0
CR to frontline therapy	52 (49)	21 (40)	NR
Relapse <12 months for patients in CR	21 (40)	11 (52)	NR

Not all percentages add to 100 due to rounding. Abbreviation: NR: not reported.

In addition to being a matter of debate for several years [4], the availability of novel agents has further increased doubts about the role and the timing of allogeneic transplantation in HL [91]. Nevertheless, the modality has a role in selected patients due to its potential for cure, particularly with reduced-intensity conditioning regimens. However, suitable donors are often not available, and haploidentical transplantation is under investigation for selected cases. Investigators from Brazil retrospectively evaluated 24 patients undergoing haploidentical transplantation for refractory or relapsed HL [109]. After a median follow-up 30 months, 2-year PFS and OS rates were 54% and 66%, respectively, with a cumulative incidence of non-relapse-related mortality of 26%, usually from infections. The authors concluded that this treatment modality is an option for patients with a relapse after ASCT, with favorable survival and relatively low risk of graft-versus-host disease.

In the Brazilian Prospective Hodgkin’s Lymphoma Registry, no specific information on primary refractoriness or relapse rates was provided, but these events were used to compute Kaplan-Meier estimates for the PFS results discussed above [3]. Moreover, of 652 patients evaluated for response to frontline treatment, 73% had a CR, 12% had unconfirmed CR, 4% had a PR, 2% had stable disease, and 9% had progressive disease. Thus, depending on the definition used, one may say that at least 11% of patients were primarily refractory (i.e., did not have at least a PR). Moreover, the overall 3-year PFS rate was 74%. Thus, one may infer that approximately 26% of patients in this series have refractory or relapsed disease with a median follow-up of 37 months [3]. At present, no results are available from the Brazilian Prospective Hodgkin’s Lymphoma Registry regarding the management of refractory or relapsed patients, whether with transplantation or novel agents, but such results are awaited.

### Availability and use of novel agents

In Brazil, BV (1.8 mg/kg intravenously every 3 weeks), nivolumab (3 mg/Kg intravenously every 2 weeks or 240 mg intravenously every 2 weeks or 480 mg intravenously every 4 weeks), and pembrolizumab (200 mg intravenously every 3 weeks or 400 mg every 6 weeks) are available as single agents for the treatment of adult patients with refractory or relapsed CHL, with slight differences in their indications and with the additional approval of pembrolizumab for pediatric patients. The evolving role of each of the currently available monoclonal antibodies leads to questions about how to make best use of each novel agent, whether alone or in combination, in frontline, as a bridge to ASCT, as consolidation after ASCT, or even as an option after failure of ASCT. Unfortunately, access to these agents is not universally available in Brazil; although healthcare is the responsibility of the federal, state and municipal governments and ensured by the constitution, the provision of care in this country is done in a dual manner [110]. A private healthcare system, available to only around 29% of the population [111], typically ensures access to all agents approved in the country following their label indications, with specific constraints for oral drugs. On the other hand, the government-funded public system suffers severe constraints and in many cases only offers access to novel therapies through judicial means [112]. It remains unclear whether such dual healthcare system explain the differences in outcomes according to SES discussed above [35]. At present, only BV is part of the treatment recommendations for HL in the public healthcare system, with indications as post-ASCT consolidation or relapse or refractoriness after ASCT [113]. However, at the time of this writing, reimbursement is not yet sufficient to ensure wide use of BV in our public healthcare system.

### Balancing efficacy, safety, and tolerability

The history of clinical trials for HL is one of the models of success in the treatment of cancer. As a result of progressive developments over the years, the balance between efficacy and safety from the use of chemotherapy and radiotherapy, particularly with regard to long-term toxicity in younger patients, has become one of the key concerns on the part of experts in this disease [114, 115]. Therefore, it is important to assess patient and physician preferences in the choice of treatment for patients with CHL. In an assessment of preferences in Europe, 5-year PFS and OS rates were the most important treatment attributes to patients choosing frontline therapy, whereas the importance of efficacy and safety attributes varied among physicians according to patient profiles [116]. A similar emphasis on efficacy on the part of patients was elicited in a US survey [117]. We are not aware of similar surveys among patients with refractory or relapsed disease, but results from the GHSG indicate that survivors of HL frequently express concern about recurrence and late toxicity from treatment [118].

Similar considerations regarding the balance between efficacy and safety also apply to novel agents, which are now changing the treatment algorithm in CHL both in the frontline and in the refractory/relapsed setting. To our knowledge, there are no published results of surveys regarding patient or physician preferences for novel agents, in reference to their risk-benefit profiles. Therefore, at present indirect comparisons need to be made for such an assessment. Table 2 presents selected efficacy and safety results from clinical trials of single-agent BV and CPIs for patients with refractory or relapsed CHL [14, 15, 57–60, 62, 72, 73, 75–77]. The accumulated experience with BV thus far suggests that the most specific adverse events are peripheral neuropathy and neutropenia, even though other, less specific events are usually more common and include fatigue, weight loss, fever, abdominal pain, stomatitis, nausea/vomiting, diarrhea, constipation, upper respiratory tract infection, anemia, and lymphopenia [119]. Peripheral neuropathy is a frequent concern in patients receiving BV, but in most cases it is of grade 1 or 2, and with dose adjustments it tends to improve or resolve over time in up to 90% of patients [62, 119, 120]. Of note, quality of life decreases were modest when BV was compared with placebo as consolidation among patients at high risk of relapse after auto-HSCT [121]. Regarding the accumulated experience with nivolumab in CHL, the most specific adverse events are colitis or diarrhea, pneumonitis, hypothyroidism, and infusion-related reactions, but more frequent events include fatigue, fever, musculoskeletal pain, rash, nausea, pruritus, cytopenias, liver-function abnormalities, and increased lipase [122]. A somewhat similar profile of adverse events is expected with pembrolizumab in CHL, given the similar mechanism of action and association with immune-mediated phenomena [123].

Although no definitive conclusions can be drawn from such indirect comparisons, and given the existence of only interim efficacy data from direct comparison between BV and pembrolizumab [15], the current literature suggests that novel agents have somewhat distinct safety profiles, and that balancing risks and benefits from these agents largely depends on patient characteristics and previous therapy, physician preference and experience, and drug availability [120]. Moreover, the results from real-life studies, discussed above, suggest that novel agents can generally be administered to patients with refractory or relapsed CHL based on their current indications and with expected results that are similar to those from clinical trials, particularly if recommended precautions are followed regarding the recognition and management of toxicity.

### CONCLUSIONS

Refractory or relapsed CHL continues to represent a therapeutic challenge, but the introduction of novel agents seems to have change the outlook for patients over the last decade. The therapeutic landscape is undergoing profound changes brought about by these agents, and their interplay with autologous and allogeneic transplantation continues to evolve. The management of patients with refractory or relapsed CHL in the Brazilian healthcare setting is constrained by inherent characteristics of this system, and a similar situation may be found in other countries. In the attempt to balance efficacy, safety and tolerability of salvage therapy, practicing physicians can rely on clinical trials and on results from real-life studies with novel agents. The accumulated literature thus far suggests that BV and CPIs are all active in refractory or relapsed CHL, and that they have somewhat distinct safety profiles and slightly differing indications. As a result, patient characteristics and previous therapy, physician preference and experience, and drug availability should dictate treatment choice for refractory or relapsed CHL.
